# Genetic Risk Score Increased Discriminant Efficiency of Predictive Models for Type 2 Diabetes Mellitus Using Machine Learning: Cohort Study

**DOI:** 10.3389/fpubh.2021.606711

**Published:** 2021-02-17

**Authors:** Yikang Wang, Liying Zhang, Miaomiao Niu, Ruiying Li, Runqi Tu, Xiaotian Liu, Jian Hou, Zhenxing Mao, Zhenfei Wang, Chongjian Wang

**Affiliations:** ^1^Department of Epidemiology and Biostatistics, College of Public Health, Zhengzhou University, Zhengzhou, China; ^2^School of Information Engineering, Zhengzhou University, Zhengzhou, China

**Keywords:** type 2 diabetes, risk prediction, genetic risk score, machine learning, cohort study

## Abstract

**Background:** Previous studies have constructed prediction models for type 2 diabetes mellitus (T2DM), but machine learning was rarely used and few focused on genetic prediction. This study aimed to establish an effective T2DM prediction tool and to further explore the potential of genetic risk scores (GRS) via various classifiers among rural adults.

**Methods:** In this prospective study, the GRS for a total of 5,712 participants from the Henan Rural Cohort Study was calculated. Cox proportional hazards (CPH) regression was used to analyze the associations between GRS and T2DM. CPH, artificial neural network (ANN), random forest (RF), and gradient boosting machine (GBM) were used to establish prediction models, respectively. The area under the receiver operating characteristic curve (AUC) and net reclassification index (NRI) were used to assess the discrimination ability of the models. The decision curve was plotted to determine the clinical-utility for prediction models.

**Results:** Compared with the individuals in the lowest quintile of the GRS, the HR (95% CI) was 2.06 (1.40 to 3.03) for those with the highest quintile of GRS (*P*_trend_ < 0.05). Based on conventional predictors, the AUCs of the prediction model were 0.815, 0.816, 0.843, and 0.851 via CPH, ANN, RF, and GBM, respectively. Changes with the integration of GRS for CPH, ANN, RF, and GBM were 0.001, 0.002, 0.018, and 0.033, respectively. The reclassifications were significantly improved for all classifiers when adding GRS (NRI: 41.2% for CPH; 41.0% for ANN; 46.4% for ANN; 45.1% for GBM). Decision curve analysis indicated the clinical benefits of model combined GRS.

**Conclusion:** The prediction model combined with GRS may provide incremental predictions of performance beyond conventional factors for T2DM, which demonstrated the potential clinical use of genetic markers to screen vulnerable populations.

**Clinical Trial Registration:** The Henan Rural Cohort Study is registered in the Chinese Clinical Trial Register (Registration number: ChiCTR-OOC-15006699). http://www.chictr.org.cn/showproj.aspx?proj=11375.

## Introduction

Type 2 diabetes mellitus (T2DM) is a global health threat (1). There has been an abrupt ascent of this disease worldwide, particularly in developing areas (2). Even though low- and middle-income countries account for 87% of diabetes-pertinent deaths, they only account for 35% of diabetes-pertinent health expenditure worldwide (2). Risk prediction tools have been developed as a prevention strategy to identify individuals at high risk of T2DM in specific populations (3–6). However, this work has largely been conducted using classical statistical models with strict and limited assumptions such as logistic and Cox regression and only include common conventional T2DM risk factors. Recently, machine learning methods have been used to solve these prediction problems with considerable discrimination (7–9), and could provide an appropriate approach to data involving interrelated and complex structures.

Genetic factors as well as common conventional risk factors are contributed to the causes of T2DM and have led to much interest in disease prediction (10). Along with the rise of the genome-wide associations study (GWAS), a handful of significant single nucleotide polymorphisms (SNPs) associated with T2DM have been identified in recent years (11, 12). The SNPs are often massed as a genetic risk score (GRS) using genetic information (13–17). However, the practical use of genetic variation is still controversial (18) and is rarely used as a practical prediction factor in clinical applications. Genetic predisposition presents disparity among contrasting ethnicities (10) and recent studies for genome analysis have largely involved non-Asian populations (14–17) with few cohort studies conducted (13).

It is therefore imperative to undertake a study of genetic prediction for T2DM in individuals from an Asian cohort. Furthermore, low socioeconomic status is associated with an increase in T2DM risk (19, 20) and few studies have performed T2DM prediction in economic and resource-limited areas in China. Therefore, this study aimed to establish an effective T2DM prediction tool and further evaluate the potential of GRS in T2DM prediction among a rural Chinese population from a 3-years follow-up cohort study using machine learning methods.

## Materials and Methods

### Study Design and Participants

The Henan Rural Cohort Study was a large-scale study with the long-term purpose examining the natural histories of chronic non-communicable diseases (NCDs) in the Henan Province of China. Detailed descriptions of study design and eligibility criteria have been published elsewhere (21–23). Participants who were permanent residents aged 18–79 years old were recruited. A subpopulation of this cohort comprising 8,268 individuals, whose venous blood samples were available for DNA extraction and T2DM SNPs selection, was included. The baseline survey was performed in 2015 and a follow-up survey was performed in 2018. The standardized questionnaire and physical examination were carried out during both baseline and follow-up surveys by specially trained staff.

For this study, we focused on participants who were available for the known outcome, complete predictors, and genotype data. This resulted in 5,712 individuals after the exclusion of prevalent T2DM cases at baseline (*n* = 764), unknown incident T2DM (*n* = 686), and incomplete epidemiology and genotype data (*n* = 1,106). With the 3-years follow-up time, a total of 5,712 participants was randomly split into the training (70%, *n* = 3,998) and test datasets (30%, *n* = 1,714) to establish models and evaluate prediction performance, respectively.

This study was approved by the Zhengzhou University Life Science Ethics Committee [Code: [2015] MEC (S128)]. Written informed consent was obtained from all participants.

### Variables and Outcome Collection

During the baseline survey, information including demographic characteristics, lifestyle, personal history of diseases, and parental history of diabetes was collected by face to face interviews. Venous blood samples were obtained after 8-h overnight fasting and sent with cold-chain transportation for lipid profile, fasting plasma glucose (FPG) measurements, and DNA extraction. The SNP genotype was performed using a custom-by-design SNPscan^TM^ Kit (Genesky Biotechnologies Inc., Shanghai, China). Anthropometric measurements including height, weight, waist circumference (WC), and resting blood pressure were conducted at least twice using calibrated instruments. Hypertension was considered as systolic blood pressure (SBP) > 140 mmHg, and/or diastolic blood pressure (DBP) > 90 mmHg, or the current use of antihypertensive medication. Dyslipidemia was identified as having one or more of the following conditions: total cholesterol (TC) ≥6.2 mmol/L (240 mg/dL); triglycerides (TG) ≥ 2.3 mmol/L (200 mg/dL); high-density lipoprotein cholesterol (HDL-C) ≤ 1.0 mmol/L (40 mg/dL); low-density lipoprotein cholesterol (LDL-C) ≥4.1 mmol/L (160 mg/dL) or use of lipid-lower drugs in recent days. Physical activity was defined in previous literature (24). Participants whose mother or father had diabetes were considered as having a parental history of diabetes.

New-onset type 2 diabetes was the primary outcome in this study, defined as self-reported new physician diagnosis of T2DM with current use of oral hypoglycemic medications or insulin, or FPG ≥ 7.0 mmol/L during the follow-up period.

### SNPs Selection and GRS

In this study, a total of 17 SNPs identified in GWAS and replicated within our cohort individuals were used to construct a weighted genetic risk score, which involved of rs10811661, rs10886471, rs1359790, rs1436955, rs17584499, rs2237892, rs2299620, rs2383208, rs4712523, rs5945326, rs6467136, rs7041847, rs7403531, rs7754840, rs7756992, rs831571, and rs9470794. The detailed filters were applied using the following procedure: first, we replicated 17 T2DM-associated SNPs reported by previous GWAS in a pilot case-control study among our population. Secondly, a natural population derived from the Henan Rural Cohort Study was the target group (as mentioned before) to explore the association of 17 SNPs with incident T2DM risk during 3-years follow-up.

The cumulative effects of 17 SNPs were accessed by calculating a GRS. In this study, a weighted GRS was developed using the risk effect from the Cox proportional hazards (CPH) regression model. Considering the assumption that the effect size is not equal across risk alleles, the time of T2DM onset was used to estimate time-specific risks of T2DM for each participant. Derived from the survival analysis model, the weighted GRS was therefore designed to predict the time-related risk of disease (25). The score was calculated as below: Firstly, each SNP was coded as 0/1/2 according to the number of the risk alleles. Secondly, the weights of the SNPs derived from the CPH regression [the Ln of hazard ratios [HRs]] for T2DM were obtained. Then GRS was calculated using the following formula: $GRS=\sum_{i=1}^{k}Ln\left(HR_{i}\right)SNP_{i}$, where *k* means the number of SNPs included in this study; *Ln(HR*_*i*_*)* means the Ln of the hazard ratio of the *i*-th SNP; *SNP*_*i*_ means the risk allele dosages (0/1/2) of the *i*-th SNP. A list of the information of SNPs in this study was provided in Supplementary Table 1.

### Prediction Model Construction

Non-genetic T2DM candidate predictors, consisting of age, gender (1 = man; 0 = woman), FPG, BMI, HDL-C, TG, hypertension (1 = with hypertension; 0 = without hypertension), parental history of diabetes (1 = with history; 0 = without history), physical activity (1 = high level; 0 = low level), WC, history of dyslipidemia (1 = with history; 0 = without history), DBP, and SBP, are identified in previous studies (4, 5), while GRS was considered as the additional genetic predictor. The training dataset was used to select the candidate predictors. Given the time window between the measurement of predictor levels and the occurrence of the developed T2DM, the CPH regression could be suitable for the development of the basic models (26). Thus, CPH was chosen as the general statistical model to select the predictors and predict T2DM during 3-years follow-up. Predictors were selected by CPH as following: (1) a univariate CPH regression was used to analyze the T2DM risk of all candidate-conventional-factors in training dataset; (2) we fitted the significant variables (*P* ≤ 0.05) from univariate analysis into multivariate CPH regression models using stepwise selection, with *P* ≤ 0.05 for inclusion and *P* > 0.10 for exclusion. For each iteration, the significant variable (*P* ≤ 0.05) with the lowest *P*-value was included. After adding a variable, the insignificant (*P* > 0.10) variable was excluded until all variables were significant.

After selected the predictors, we used the training data to determine the optimal parameters of each prediction model. We then examined performance in the test dataset using CPH, and machine learning methods. In the training procedure, the 10-fold cross-validation was conducted and the Grid search was applied to select the hyper-parameters. To ensure the stability of the parameter, we repeated the process 100 times. We used the area under the receiver operating characteristic curve (AUC) as an indicator to evaluate the model performance. The parameter leading to the highest AUC of the model was considered to be the optimal parameter. Finally, the models with hyper-parameters were used to evaluate the performances in the test dataset. The work flow of the model construction was shown in Supplementary Figure 1.

For CPH model construction, the conventional prediction model was derived using the selected risk factors above. The effects of all predictors were from the fitted CPH regression in the training dataset. Then prediction models were developed in the test dataset to predict onset T2DM risks for each participant. The conventional + GRS model was constructed using a similar procedure. The predictor-related coefficients in CPH models are shown in Supplementary Table 4.

Here, machine learning methods named ANN, RF, and GBM were used to construct and test prediction models. Published studies have demonstrated the ANN (27), RF (28), and GBM (29) have considerable performances with medical data in disease prediction. Our previous study indicated that the predictive abilities for these three classifiers were the top three (30). Using the Grid search method, the optimal parameters were determined to build models Optimization details and the final parameters are shown in Supplementary Table 5.

ANN is one of the non-linear regression models designed to simulate the structure and function of the human brain to manipulate information using the characteristics of self-adaption, self-organization, and self-learning. Multiple neurons are contained in the input, hidden, and output layers of ANN. Directed arcs with adjustable coefficients connect the layers. ANN trains the input information to change the coefficients in the transfer function, and then get one output, which can cognize the relationships patterns of data imitating the neural frame of human.

RF is one of the ensemble learning methods with randomly produced independent decision trees. Each decision tree bases on a randomly selected subset and selects the optimal attribute for partitioning. The randomly selected subset using bagging introduces amounts of random trees to get an ensemble of classification. Then the average classification is selected by choosing the majority of votes. Using the bagging theory, RF seems to be an accurate and robust tool without overfitting.

GBM is an additive algorithm and boosting technique is applied to construct weaker classifiers into multiple iterations to get an improved and stronger model. In each iteration, the same training set data is used to fit various classifiers (weak classifiers), and repeated progress is conducted to get an enhanced model that overcomes the shortcomings of previous weaker classifiers based on the residual. The progress of model-optimized iteration provides new base-learners with more accuracy.

### Statistical Analysis

Data for numerical variables were expressed as mean ± standard deviation and compared by Student's *t*-test, while categorical variables were expressed in frequency and percentage (*n*, %) and compared by chi-squared test. CPH regression model was used to estimate the associations of GRS. The dose-response relationship between GRS and T2DM was explored.

For the performance of the prediction model, AUC was used to assess model discrimination, while the Brier Score (BS, mean square of the deviation between predicted and observed risks) was listed to evaluate the calibration. Higher AUC and lower BS indicate better discrimination and calibration, respectively. The incremental predictive value of GRS for an additional predictor to the conventional risk prediction model was evaluated by the change of AUC and risk reclassification analysis. DeLong's method was used to compare the difference of AUCs between prediction models (31). The continuous net reclassification improvement (NRI) was calculated to detect model improvement after adding genetic markers (32). NRI equals the sum of the percentage of patients who were correctly reclassified and the percentage of disease-free individuals who are correctly reclassified. Bootstrapping was used to estimate the 95% confidence intervals (CIs) of NRI with 1000 replications. Higher NRI indicates better reclassification. For the clinical impact of GRS, decision curve analysis was applied to calculate the net benefit by comparing the conventional and conventional + GRS models (33).

Statistical analysis was implemented by Python (version 3.7.3) and R software (version 3.6.1). All tests were two-sided (*P* < 0.05).

## Results

### Baseline Characteristics

The baseline characteristics of the subjects in the total dataset are outlined in Table 1. A total of 324 individuals developed T2DM (incidence, 5.67%) among 5,712 subjects. Compared with participants without developed T2DM, the developed T2DM patients were older and heavier, and a larger proportion had a history of hypertension and dyslipidemia with not optimistic blood pressure and lipid profiles (higher SBP, DBP, TG, lower HDL-C), as well as higher FPG (all *P* < 0.05). However, the individual distributions were different in training and test datasets (Supplementary Table 2).

**Table 1:** Baseline characteristics of subjects in the cohort study.

**Parameters**	**T2DM (*n =* 324)**	**NON-T2DM (*n =* 5,388)**	***P*-value**
**Mean** **±** **SD**			
Age (year)	53.25 ± 10.46	51.04 ± 12.12	0.018
FPG (mmol/L)	5.94 ± 0.61	5.31 ± 0.53	<0.001
WC (cm)	89.63 ± 10.9	82.57 ± 9.93	<0.001
BMI (kg/m^2^)	26.60 ± 3.82	24.51 ± 3.50	<0.001
HDL-C (mmol/L)	1.11 ± 0.23	1.18 ± 0.26	<0.001
LDL-C (mmol/L)	2.63 ± 0.80	2.60 ± 0.74	0.122
TG (mmol/L)	1.95 ± 0.97	1.49 ± 0.77	<0.001
TC (mmol/L)	4.64 ± 0.97	4.45 ± 0.89	<0.001
SBP (mmHg)	135.45 ± 23.5	125.34 ± 20.04	<0.001
DBP (mmHg)	83.68 ± 12.12	78.42 ± 11.51	<0.001
**Frequency (%)**			
Man	108 (33.33)	1,966 (36.49)	0.251
Physical activity	190 (58.64)	2,818 (52.30)	0.026
Dyslipidemia	192 (59.30)	2,266 (42.06)	<0.001
Hypertension	186 (57.41)	1,648 (30.59)	<0.001
Parental history of diabetes	36 (11.11)	274 (5.09)	<0.001

*SD, standard deviation; T2DM, type 2 diabetes mellitus; FPG, fasting plasma glucose; WC, waist circumference; BMI, body mass index; HDL-C, high-density lipoprotein cholesterol; LDL-C, low-density lipoprotein cholesterol; TG, triglycerides; TC, total cholesterol; SBP, systolic blood pressure; DBP, diastolic blood pressure*.

### Analysis of the Relationship Between GRS and T2DM

**Figure 1:**
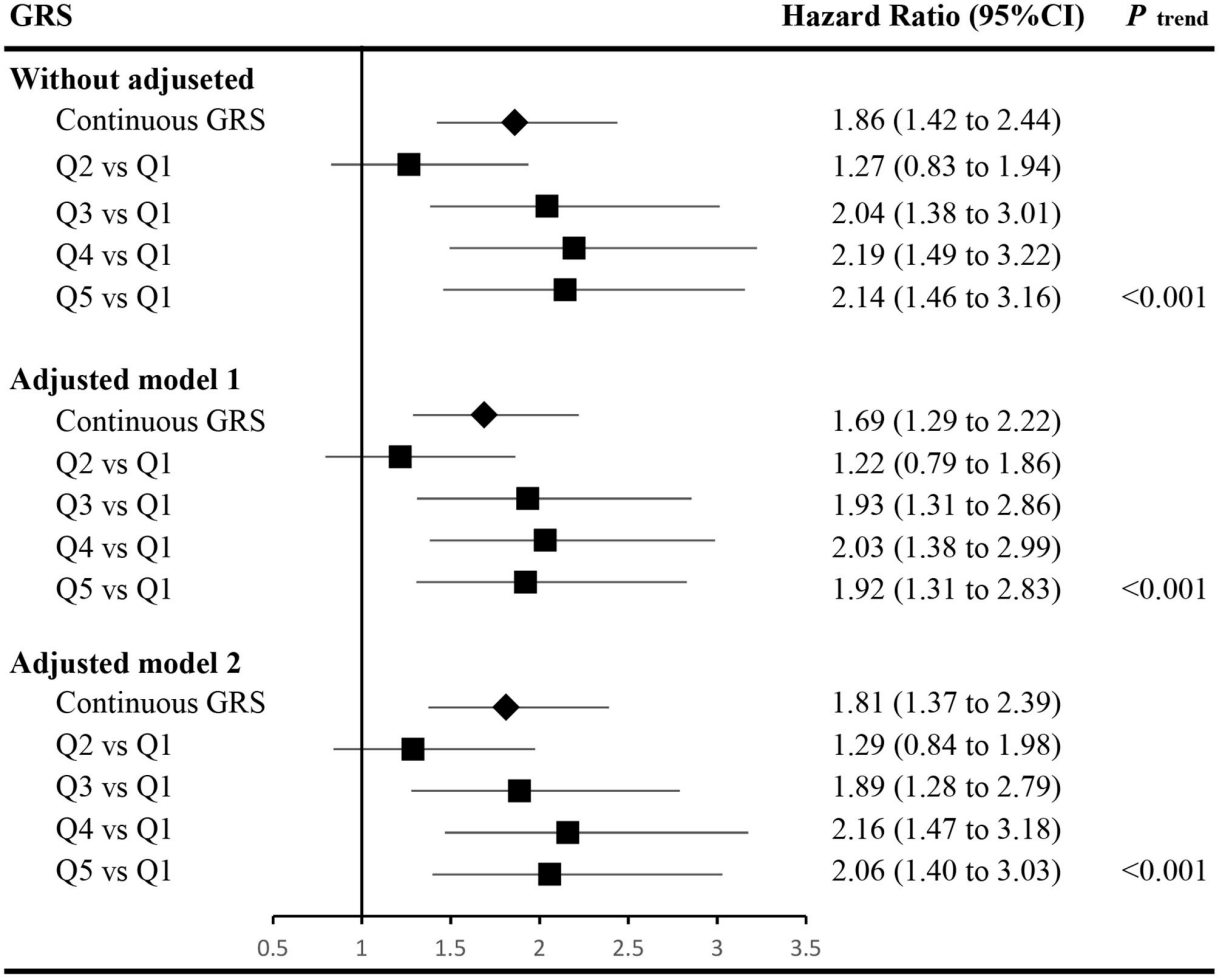
Associations between the genetic risk score and the risk of incident type 2 diabetes. The hazard function of adjusted model 1 was *h(t)*_*adjusted*−*model*−1_=*h*_0_*(t)*exp(β_GRS_*GRS+β_age_*age+β_gender_*gender+β_FPG_*FPG+β_PHOD_*PHOD), and the hazard ratios of the GRS were calculated accounting for the covariates of age, gender, fasting plasma glucose (FPG), and parental history of diabetes (PHOD). The hazard function of adjusted model 2 was *h(t)*_*adjusted*−*model*−2_=*h*_0_*(t)*exp(β_GRS_*GRS+β_age_*age+β_gender_*gender+β_FPG_*FPG+β_PHOD_*PHOD+β_WC_*WC+β_TG_*TG+β_hypertension_*hypertension), and the hazard ratios of the GRS were calculated accounting for the covariates in adjusted model 1 and waist circumference (WC), triglycerides (TG), and hypertension as well. *h*_0_*(t)* indicates the baseline hazard function. GRS, genetic risk score; FPG, fasting plasma glucose; PHOD, parental history of diabetes; WC, waist circumference; TG, triglycerides; CI, confidence interval.

The associations between GRS and incident of T2DM are displayed in Figure 1. Accounting for covariates of age, gender, FPG, WC, TG, parental history of diabetes, and hypertension (adjusted model 2, detailed hazard function was expressed in Figure 1), the HR (95% confidence interval, CI) of continuous GRS was 1.81 (1.37–2.39). Compared with the lowest quintile of the GRS, the HR (95% CI) of those with the highest quintile of GRS in adjusted model 2 was 2.06 (1.40–3.03). The dose-response relationship between GRS and developed T2DM was also observed with and without adjusted (*P*_trend_ < 0.05).

### Conventional Risk Factors Selection

During univariate CPH regression in the training dataset, age, FPG, BMI, HDL-C, TG, hypertension, parental history of diabetes, physical activity, WC, history of dyslipidemia, DBP, and SBP were significantly associated with T2DM (Supplementary Table 3). Then, during the stepwise selection, FPG, TG, WC, hypertension, and parental history of diabetes were the T2DM risk factors (Supplementary Table 3), and a conventional-risk-prediction model was constructed using the predictors above.

### Comparison of Models With and Without GRS

**Figure 2:**
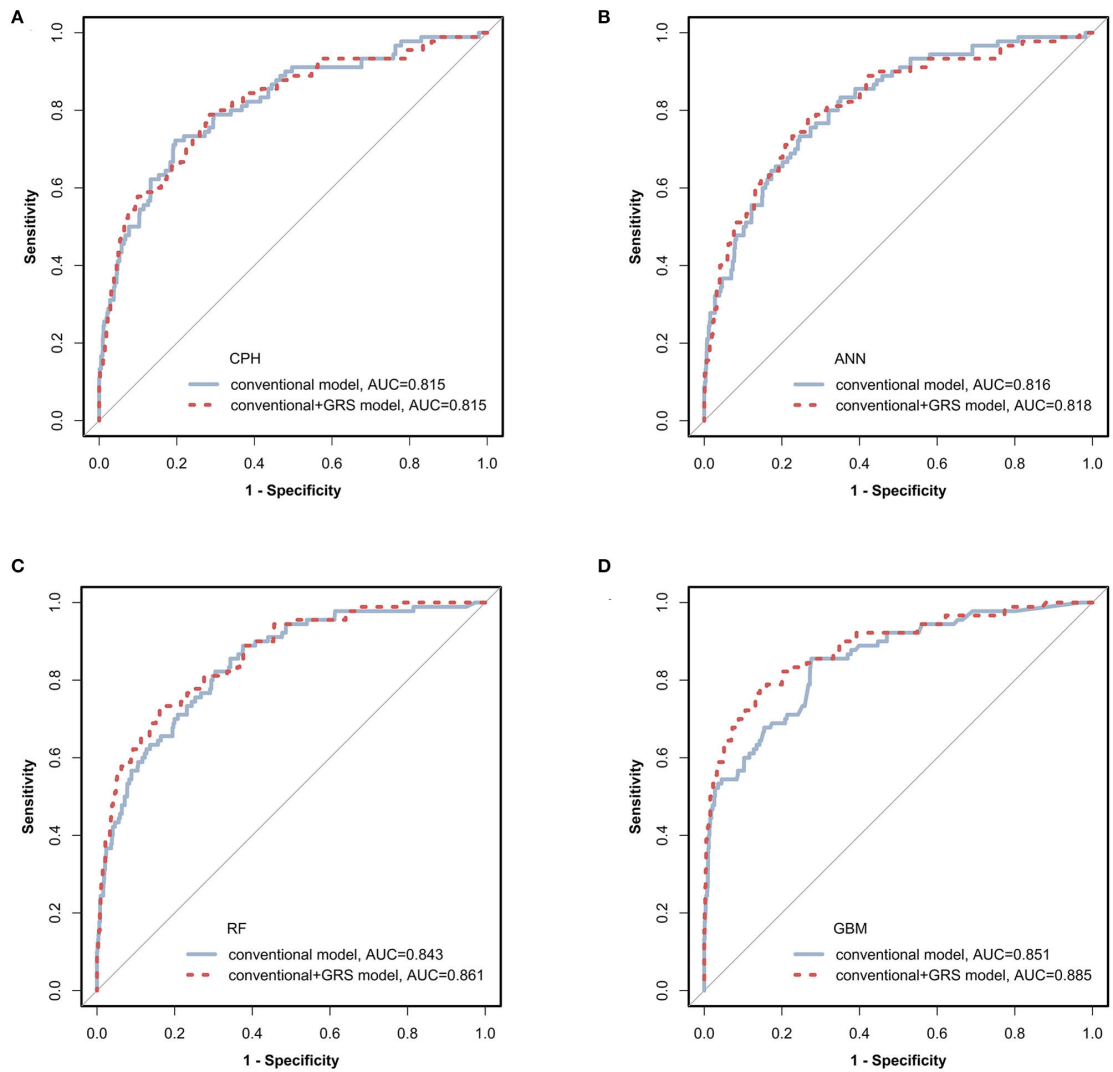
Receiver operating characteristic (ROC) curves of prediction models via four classifiers. The prediction models constructed using conventional (blue solid line) and conventional + GRS (red dashed line) predictors were compared via CPH, ANN, RF, and GBM as shown in **(A–D)**, respectively. GRS, genetic risk score; CPH, Cox proportional hazards regression model; ANN, artificial neural network; RF, random forest; GBM, gradient boosting machine.

We tested the model discriminations and calibrations of 4 classifiers in the test dataset to evaluate the performance of models (Table 2 and Figure 2). For conventional risk prediction models, the AUCs (95% CI) were 0.815 (0.795–0.833), 0.816 (0.797–0.834), 0.843 (0.825–0.860), and 0.851 (0.834–0.868) using CPH, ANN, RF, and GBM methods, respectively. Calibrations were considerable and the BSs of CPH, ANN, RF, and GBM were 0.053, 0.044, 0.041, and 0.033, respectively.

**Table 2:** Performance of type 2 diabetes risk models via various classifiers.

**Classifier**	**Prediction model^**a**^**	**Discrimination**		**BS**	**NRI (95% CI)^**c**^ %**
		**AUC (95% CI)**	**ΔAUC (95% CI)^**b**^**		
CPH	Conventional model	0.815 (0.795 to 0.833)	-	0.053	-
	Conventional + GRS model	0.815 (0.796 to 0.833)	0.001 (−0.009 to 0.010)	0.133	**41.2 (27.8 to 54.1)**
ANN	Conventional model	0.816 (0.797 to 0.834)	-	0.044	
	Conventional + GRS model	0.818 (0.799 to 0.836)	0.002 (−0.015 to 0.019)	0.045	**41.0 (25.1 to 52.7)**
RF	Conventional model	0.843 (0.825 to 0.860)	-	0.041	-
	Conventional + GRS model	0.861 (0.844 to 0.877)	**0.018 (0.002 to 0.034)**	0.040	**46.4 (35.2 to 57.6)**
GBM	Conventional model	0.851 (0.834 to 0.868)	-	0.033	-
	Conventional + GRS model	0.885 (0.869 to 0.899)	**0.033 (0.001 to 0.065)**	0.033	**45.1 (18.0 to 57.7)**

*Bold values represent P < 0.05. CPH, Cox proportional hazards regression model; ANN, artificial neural network; RF, random forest; GBM, gradient boosting machine; GRS, genetic risk score; AUC, the area under receiver operating characteristic curve; BS, brier score; NRI, net reclassification improvement; CI, confidence interval*.

a *Conventional model included FPG, WC, TG, parental history of diabetes, and hypertension*.

b *ΔAUCs were the differences of AUCs among conventional-genetic-combined models and conventional model*.

c *NRI showed the reclassification of the prediction models with genetic risk scores compared to conventional model*.

The prediction performances of the conventional + GRS models are displayed in Table 2 and Figure 2. For the CPH classifier, the AUC of the prediction model with GRS was not significantly increased (the change of AUC: 0.001, 95% CI: −0.009 to 0.010) and the BS value was also not better. However, reclassification was improved after accounting for the GRS in the prediction model (NRI: 41.2%, 95% CI: 27.8–54.1%).

Machine learning methods showed excellent performance of the prediction models with the addition of GRSs (Table 2 and Figure 2), especially for GBM (Supplementary Table 6). When adding the GRS to the basic model, the changes of AUCs went up to 0.002, 0.018, and 0.033 based on ANN, RF, and GBM, respectively. Notably, using RF and GBM, significant improvements of AUC were observed (*P* = 0.023 for RF, *P* = 0.041 for GBM) and the combined models presented better BSs. For reclassification analysis, the NRIs (95% CI) were 41.0% (25.1–52.7%), 46.4% (35.2–57.6%), and 45.1% (18.0–57.7%) for ANN, RF, and GBM (all *P* < 0.05).

### Clinical Impact of GRS

**Figure 3:**
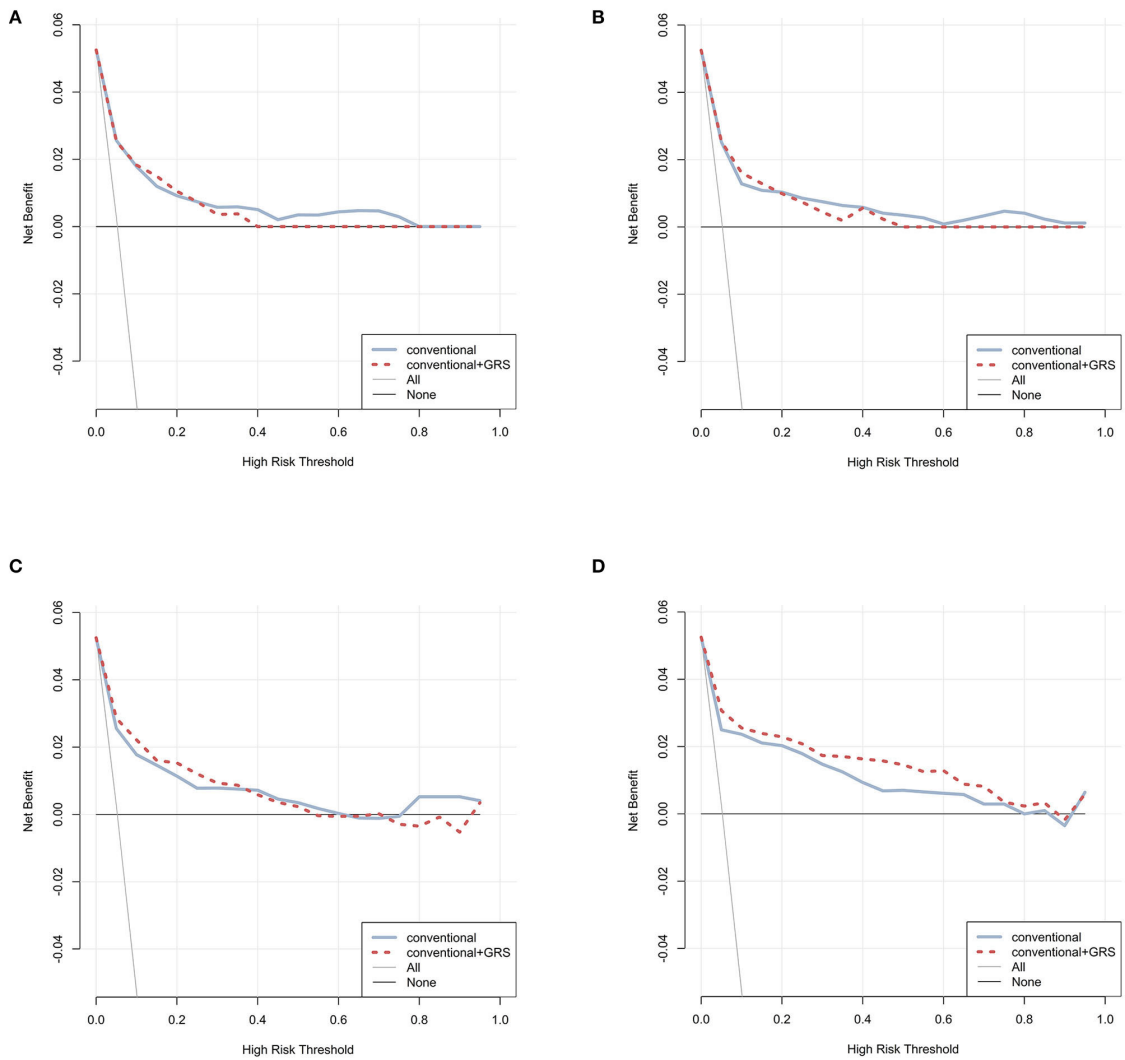
Decision curve analysis for prediction models via four classifiers. The net benefits of the prediction models using CPH, ANN, RF, and GBM were plotted in **(A–D)**, respectively. The blue solid line performed the prediction model constructed using conventional predictors. The red dashed line performed the prediction model constructed using conventional + GRS predictors. The gray solid line performed the assumption that all individuals were in the presence of intervention. The black solid line performed the assumption that all individuals were in the absence of intervention. GRS, genetic risk score; CPH, Cox proportional hazards regression model; ANN, artificial neural network; RF, random forest; GBM, gradient boosting machine.

Decision curves were plotted for the conventional and conventional + GRS models using test data (Figure 3). For CPH, ANN, and RF classifiers, the decision curves crossed repeatedly for models with and without GRS. Conversely, within almost all ranges of the threshold probability, the net benefit of the model combined GRS was higher than that of the basic conventional model for the GBM classifier.

## Discussion

The present large-scale cohort study among a Chinese rural population calculated a GRS, assembling the information of 17 identified susceptible SNPs for T2DM, and evaluated their additional prediction performances by applying CPH and machine learning methods. In this study, a basic prediction model consisting of common conventional T2DM risk factors was established and showed impressive and robust performance using machine learning methods. With the addition of genetic information to the basic model, significant improvement was observed, which means more accurate classification for the patients and non-T2DM individuals. These results indicate that the use of genetic information might help promote the practical prevention of T2DM. Therefore, using a prediction tool with genetic information in diabetes screening could help select high-risk populations and enable interventions to prevent the disease.

Our analysis of the prediction model selected five predictors (FPG, TG, WC, hypertension, and parental history of diabetes), had robust and considerable discrimination (AUC ≥0.815 for all classifiers) and the AUC combined GRS came up to 0.851 based GBM. Compared with the established T2DM risk assessment tools (3–6), similar factors in the prediction model were found. One widely adopted incident T2DM risk engine was established by Wilson et al. based on the American population from Framingham Offspring Study (5). Applying FPG, TG, HDL-C, BMI, hypertension, and parental history of diabetes in prediction, the AUC of the model reached 0.85. Julia et al. updated the QDiabetes-2018 risk prediction algorithm and the AUC of the model was 0.878 for women and 0.855 for men (3). However, there were more than 10 predictors in this sophisticated risk prediction algorithm. For Chinese rural individuals, our prediction model using common conventional risk factors might be an effective and durable tool to distinguish T2DM patients and non-patients.

As an aggregation to interpret genetic predisposition, the additional predictive value of GRS upon the basic model with only conventional factors was significantly considerable, especially using machine learning. Our results demonstrated that adding the genetic risk score of GRS caused an impressive and significant increase of AUC from 0.851 to 0.885, based on GBM. Notably, GRS accounted for the improved reclassification and the considerable NRIs were 41.2%, 41.0%, 46.4%, and 45.1% for CPH, ANN, RF, and GBM, respectively, which suggested that GRS helped correct risk stratification to screen adults at high risk for T2DM. Previous work also demonstrated the potential of GRS among people from Asia (13), the UK (17), and Finland (34). Genetic information offers subtler and more unique characters for each individual in T2DM prediction compared with common epidemiological data. The predicted value of the GRS and the clinical use of genetic markers warrants further larger-scale internationally-cooperative investigations.

Machine learning classifiers in this analysis presented a higher performance compared with the classical CPH model, especially via GBM. Data mining methods, which interpret the nonlinear associations and high dimensional of a large number of variables without model assumption, might be appropriate as a useful and robust tool in T2DM risk prediction. Several studies have demonstrated that machine learning methods showed higher predictive performance in risk prediction (7, 8, 35). Accurate and robust prediction of genome data, which have complex traits and multivariate correlation, was also conducted using data mining methods in this analysis. Previous research indicates that this technology is effective in exploring the potential of genetic markers (7–9, 18). The decision curve showed the higher net benefit of the model with GRS via GBM, which demonstrated the clinical utility of genetic information to promote decision-making for clinicians. In particular, ANN had a minor improvement in discrimination compared with CPH (differences < 0.005). However, higher AUCs were observed for RF and GBM models, which demonstrated the reproducible high performance of ensemble learning.

The strengths of this study included our analysis in which the predictive value of GRSs was evaluated among 3-years follow-up Chinese rural adults. This approach might offer supplementary evidence of the clinical utility of hereditary information in Asia and resource-limited areas. Machine learning methods were also applied to derive predictive tools of T2DM and showed considerable and robust prediction performance to explore the potential of GRS compared with the classical statistical methods. Limitations included first, the fact that there were only 17 genetic loci of T2DM for GRS construction. Nevertheless, four classifiers were employed to explore the utility of GRS and the results were consistent. Second, there was no external cohort to validate the prediction model. In our analysis, however, a total of 30% of individuals were randomly selected to test the performance of the risk prediction models (test datasets). Finally, the participants in this study were from Chinese rural areas and future studies should expand into the population with different inherited-background and economic circumstances.

## Conclusion

The present study has demonstrated that the additional predictive value of genetic risk scores for incident T2DM was significantly improved among 3-years follow-up in Chinese rural adults, especially using machine learning. With further development of data mining methods, hereditary information might offer finer and more unique risk prediction for individuals and help to classify T2DM patients and non-patients. From the perspective of prevention, the conventional factors of T2DM might be enough for general T2DM screening but with further improvement and the use of machine learning, genome information might enable more accurate and intensive predictions for precision medicine to promote clinical practice.

## Data Availability Statement

The raw data supporting the conclusions of this article will be made available by the authors, without undue reservation.

## Ethics Statement

The studies involving human participants were reviewed and approved by This study was approved by the Zhengzhou University Life Science Ethics Committee [Code: [2015] MEC (S128)]. The patients/participants provided their written informed consent to participate in this study.

## Author Contributions

CW conceived and designed the experiments. MN, RL, and RT gathered data. YW and LZ analyzed the data and drafted the manuscript. All the authors contributed to the revision process and approved the final manuscript.

## Conflict of Interest

The authors declare that the research was conducted in the absence of any commercial or financial relationships that could be construed as a potential conflict of interest.
