# Supplementary material for: Genetic Risk Score Increased Discriminant Efficiency of Predictive Models for Type 2 Diabetes Mellitus Using Machine Learning: Cohort Study
Source: Front Public Health. 2021 Feb 17;9:606711. doi: 10.3389/fpubh.2021.606711 (PMC7925839; doi:10.3389/fpubh.2021.606711)
Supplement: Supplementary file 1 [file Table_1.DOCX]

**Supplementary material list**

**Supplementary table 1** Single nucleotide polymorphisms included in the genetic risk score in this study

**Supplementary figure 1** Work flow of the model construction

**Supplementary table 2** Baseline characteristics of subjects for developing the prediction model of type 2 diabetes mellitus in training and test datasets

**Supplementary table 3** Hazard ratios with 95% confidence intervals of type 2 diabetes predictors in the training dataset

**Supplementary table 4** Coefficients of each predictor based Cox proportional hazards regression in the training dataset

**Supplementary table 5** Using Grid Search Parameter Optimization

**Supplementary table 6** Comparison of type 2 diabetes risk prediction models using four classifiers

**Supplementary table 1** Single nucleotide polymorphisms included in the genetic risk score in this study

| **SNP** | **Nearby Gene** | **CHR** | **Position (bp)** | **Risk Allele** | **No-risk Allele** | **Hazard Ratio (95%CI)^a^** | **Weight^b^** |
| --- | --- | --- | --- | --- | --- | --- | --- |
| rs10811661 | *CDKN2A/B* | 9 | 22134095 | T | C | 0.98 (0.82 to 1.18) | -0.018 |
| rs10886471 | *GRK5* | 10 | 119389891 | C | T | 1.08 (0.86 to 1.35) | 0.075 |
| rs1359790 | *SPRY2* | 13 | 80143021 | G | A | 1.13 (0.92 to 1.39) | 0.122 |
| rs1436955 | *C2CD4B* | 15 | 62112183 | C | T | 1.28 (1.03 to 1.60) | 0.249 |
| rs17584499 | *PTPRD* | 9 | 8879118 | T | C | 1.55 (1.17 to 2.05) | 0.438 |
| rs2237892 | *KCNQ1* | 11 | 2818521 | C | T | 1.24 (1.01 to 1.52) | 0.212 |
| rs2299620 | *KCNQ1* | 11 | 2818521 | C | T | 1.05 (0.86 to 1.27) | 0.045 |
| rs2383208 | *CDKN2B* | 9 | 22122076 | A | G | 1.04 (0.86 to 1.25) | 0.038 |
| rs4712523 | *CDKAL1* | 6 | 20657333 | G | A | 1.11 (0.93 to 1.34) | 0.107 |
| rs5945326 | *DUSP9* | X | 153634467 | A | G | 1.14 (0.93 to 1.38) | 0.127 |
| rs6467136 | *GCC1-PAX4* | 7 | 127524904 | G | A | 1.00 (0.80 to 1.23) | -0.005 |
| rs7041847 | *GLIS3* | 9 | 4287466 | A | G | 1.05 (0.88 to 1.26) | 0.051 |
| rs7403531 | *RASGRP1* | 15 | 38530704 | T | C | 1.00 (0.83 to 1.21) | 0.001 |
| rs7754840 | *CDKAL1* | 6 | 20661019 | C | G | 1.10 (0.92 to 1.32) | 0.096 |
| rs7756992 | *CDKAL1* | 6 | 20679478 | G | A | 1.16 (0.96 to 1.39) | 0.146 |
| rs831571 | *PSMD6* | 3 | 64023337 | C | T | 0.93 (0.77 to 1.13) | -0.068 |
| rs9470794 | *ZFAND3* | 6 | 38214822 | C | T | 1.05 (0.86 to 1.28) | 0.044 |
| *Abbreviations: SNP, single nucleotide polymorphisms; T2DM, type 2 diabetes mellitus; CHR, chromosome; CI, confidence interval.*  *^a^ Hazard ratios for SNPs were adjusted for age, gender, fasting plasma glucose, triglycerides, waist circumference, parental history of diabetes, and hypertension.*  *^b^ The weight equals the β-coefficient of the SNPs in our result (the Ln of Hazard ratios [HRs]).* | | | | | | | |

**­
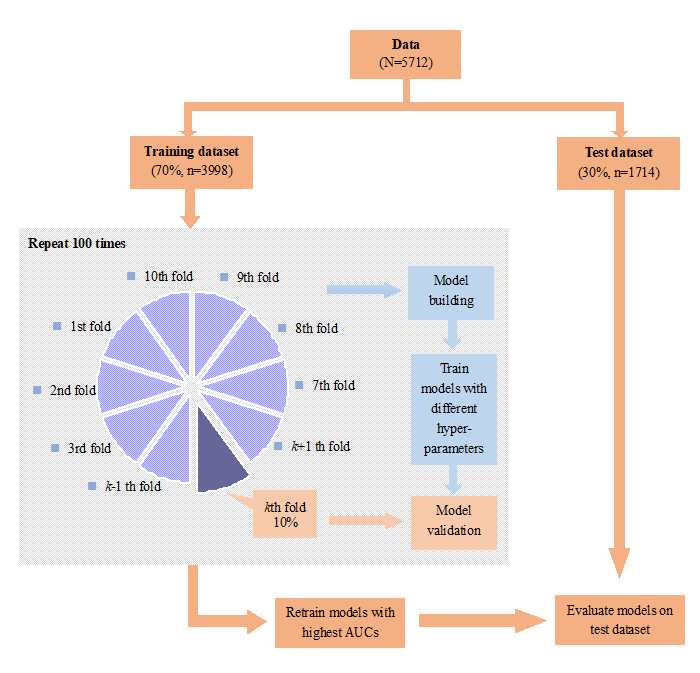
**

**Supplementary figure 1** Work flow of the model construction. Total data was randomly split into the training (70%, n=3,998) and test datasets (30%, n=1,714). We built the models and selected the optimal parameters in training dataset and the test dataset was used to evaluate the models. In training dataset, repeated 10-fold cross-validation was conducted to search the hyper-parameters for each model, and we repeated the process 100 times. The AUC was calculated to validate model’s performance, and a higher AUC was considered to be better. The model with optimal parameters was then applied to the test set to evaluate the model performance. Abbreviations: AUC, the area under receiver operating characteristic curve**.**

**Supplementary table 2** Baseline characteristics of subjects for developing the prediction model of type 2 diabetes mellitus in training and test datasets

| **Parameters** | **Training Dataset** | | |  | **Test Dataset** | | |
| --- | --- | --- | --- | --- | --- | --- | --- |
|  | **T2DM**  **(n=234)** | **NON-T2DM**  **(n=3764)** | ***P* Value** |  | **T2DM**  **(n=90)** | **NON-T2DM**  **(n=1624)** | ***P* Value** |
| **Mean ± SD** |  |  |  |  |  |  |  |
| Age (year) | 52.87 ± 10.59 | 51.15 ± 11.98 | 0.031 |  | 53.17 ± 10.10 | 50.74 ± 12.25 | 0.002 |
| FPG (mmol/L) | 5.97 ± 0.60 | 5.31 ± 0.54 | ＜0.001 |  | 5.93 ± 0.65 | 5.30 ± 0.53 | ＜0.001 |
| WC (cm) | 89.48 ± 9.92 | 82.98 ± 9.98 | ＜0.001 |  | 90.46 ± 12.8 | 82.24 ± 9.84 | ＜0.001 |
| BMI (kg/m^2^) | 26.63 ± 3.61 | 24.65 ± 3.51 | ＜0.001 |  | 26.77 ± 4.31 | 24.39 ± 3.47 | ＜0.001 |
| HDL-C (mmol/L) | 1.11 ± 0.22 | 1.17 ± 0.26 | ＜0.001 |  | 1.06 ± 0.26 | 1.18 ± 0.26 | ＜0.001 |
| LDL-C (mmol/L) | 2.69 ± 0.82 | 2.61 ± 0.74 | 0.145 |  | 2.45 ± 0.71 | 2.58 ± 0.74 | 0.539 |
| TG (mmol/L) | 2.16 ± 1.42 | 1.59 ± 1.05 | ＜0.001 |  | 2.34 ± 1.53 | 1.61 ± 1.08 | ＜0.001 |
| TC (mmol/L) | 4.75 ± 1.00 | 4.48 ± 0.91 | 0.527 |  | 4.52 ± 0.89 | 4.46 ± 0.89 | ＜0.001 |
| SBP (mmHg) | 134.85 ± 22.84 | 125.68 ± 20.21 | ＜0.001 |  | 138.04 ± 26.28 | 125.23 ± 19.90 | ＜0.001 |
| DBP (mmHg) | 83.35 ± 12.39 | 78.65 ± 11.61 | ＜0.001 |  | 85.29 ± 12.91 | 78.28 ± 11.38 | ＜0.001 |
| **Frequency (%)** |  |  |  |  |  |  |  |
| Man | 77 (32.91) | 1354 (35.97) | 0.342 |  | 31 (34.44) | 612 (37.68) | 0.537 |
| Physical Activity | 139 (59.40) | 1968 (52.28) | 0.034 |  | 51 (56.67) | 850 (52.34) | 0.424 |
| Dyslipidemia | 135 (57.69) | 1607 (42.69) | ＜0.001 |  | 57 (63.33) | 659 (40.58) | ＜0.001 |
| Hypertension | 132 (56.41) | 1167 (31.00) | ＜0.001 |  | 54 (60.00) | 481 (26.22) | ＜0.001 |
| Parental History of Diabetes | 28 (11.97) | 193 (5.13) | ＜0.001 |  | 8 (8.89) | 81 (4.99) | 0.104 |
| *Abbreviations: SD, standard deviation; T2DM, type 2 diabetes mellitus; FPG, fasting plasma glucose; WC, waist circumference; BMI, body mass index; HDL-C, high-density lipoprotein cholesterol; LDL-C, low-density lipoprotein cholesterol; TG, triglycerides; TC, total cholesterol; SBP, systolic blood pressure; DBP, diastolic blood pressure.* | | | | | | | |

| **Candidate Predictors** | **Univariate analysis** | |  | **Multivariate analysis** | |
| --- | --- | --- | --- | --- | --- |
|  | **HR (95%CI)** | ***P* Value** |  | **HR (95%CI)** | ***P* Value** |
| age | 1.01 (1.00 to 1.02) | 0.019 |  | - | - |
| gender | 1.16 (0.88 to 1.52) | 0.298 |  | - | - |
| BMI | 1.14 (1.11 to 1.18) | <0.001 |  | - | - |
| HDL-C | 0.36 (0.21 to 0.62) | <0.001 |  | - | - |
| DBP | 1.03 (1.02 to 1.04) | <0.001 |  | - | - |
| SBP | 1.02 (1.01 to 1.02) | <0.001 |  | - | - |
| Physical activity | 1.43 (1.10 to 1.86) | 0.008 |  | - | - |
| History of dyslipidemia | 1.78 (1.38 to 2.31) | <0.001 |  | - | - |
| FPG | 9.34 (7.32 to 11.93) | <0.001 |  | 7.67 (5.95 to 9.88) | <0.001 |
| TG | 1.35 (1.25 to 1.45) | <0.001 |  | 1.16 (1.06 to 1.26) | 0.001 |
| Hypertension | 2.88 (2.22 to 3.72) | <0.001 |  | 1.63 (1.24 to 2.15) | <0.001 |
| Parental History of Diabetes | 2.38 (1.60 to 3.53) | <0.001 |  | 2.25 (1.51 to 3.34) | <0.001 |
| Waist circumference | 1.06 (1.05 to 1.07) | <0.001 |  | 1.03 (1.02 to 1.04) | <0.001 |
| *Abbreviations: FPG, fasting plasma glucose; BMI, body mass index; HDL-C, high-density lipoprotein cholesterol; TG, triglycerides; SBP, systolic blood pressure; DBP, diastolic blood pressure; HR, hazard ratio; CI, confidence interval.* | | | | | |

**Supplementary table 3** Hazard ratios with 95% confidence intervals of type 2 diabetes predictors in the training dataset

|  | **conventional model** | **conventional + GRS model** |
| --- | --- | --- |
| FPG | 2.037 | 2.027 |
| TG | 0.147 | 0.146 |
| Hypertension | 0.490 | 0.503 |
| PHOD | 0.809 | 0.808 |
| WC | 0.029 | 0.030 |
| GRS | - | 0.767 |
| Hazard function | *h(t)*=*h_0_(t)*exp(2.037**FPG*+0.147**TG*  +0.490**hypertension*  +0.809**PHOD*+0.020**WC*) | *h(t)*=*h_0_(t)*exp(2.027**FPG*+0.146**TG*  +0.503**hypertension*  +0.808**PHOD*+0.030**WC*  +0.767**GRS*) |
| *h_0_(t) indicated the baseline hazard function.*  *Abbreviations: FPG, fasting plasma glucose; TG, triglycerides; PHOD, parental history of diabetes; WC, waist circumference; GRS, genetic risk score.* | | |

**Supplementary table 4** Coefficients of each predictor based Cox proportional hazards regression in the training dataset

**Supplementary table 5** Using Grid Search Parameter Optimization

| Algorithm | Parameters tuned | Grid Search Script | AUC range | Optimal performance |
| --- | --- | --- | --- | --- |
| Artificial neural networks (ANN) | hidden_layer_sizes=np.arange(1, 12)  max_iter(1000, 2000, 3000)  learning_rate_init (0.0001, 0.001, 0.01,0.1)  activation{‘identity’, ‘logistic’, ‘tanh’, ‘relu’} | scoring = {'AUC': 'roc_auc'}  hidden_layer_sizes=np.arange(1, 12)  max_iter=[1000, 2000, 3000]  learning_rate_init=[0.0001, 0.001, 0.01,0.1]  activation=[‘identity’, ‘logistic’, ‘tanh’, ‘relu’]  param_grid = [ {' hidden_layer_sizes ': hidden_layer_sizes,  ' max_iter ': max_iter , ' learning_rate_init ': learning_rate_init,  ' activation ': activation} ]  clf = GridSearchCV(MLPClassifier (), param_grid, scoring=scoring, cv=10, refit='AUC', return_train_score=True) | **conventional model:**  0.5-0.858  **conventional + GRS model:**  0.62-0.865 | **conventional model:**  0.816  hidden_layer_sizes=11  max_iter=2000  **conventional + GRS model:**  0.818  hidden_layer_sizes=6,3  max_iter=2000  learning_rate_init=0.001  activation= ' tanh ' |
| Random Forest(RF) | n_estimators =np.arange(50, 100, 10)  max_depth=np.arange(1, 10) | scoring = {'AUC': 'roc_auc'}  param_grid = [ {n_estimators=np.arange(50, 100, 10), max_depth=np.arange(1, 10) } ]  clf = GridSearchCV(RandomForestClassifier (), param_grid, scoring=scoring, cv=10, refit='AUC', return_train_score=True) | **conventional model:**  0.823-0.857  **conventional + GRS model:**  0.836-0.870 | **conventional model:**  0.843  n_estimators=80  max_depth=4  **conventional + GRS model:**  0.861  n_estimators=60  max_depth=5 |
| Gradient boosting machine (GBM) | n_estimators=np.arange(10, 100, 10)  max_depth=np.arange(1, 10)  min_samples_leaf=np.arange(1, 7) | scoring = {'AUC': 'roc_auc'}  param_grid = [ { n_estimators=np.arange(10, 100, 10), max_depth=np.arange(1, 10), min_samples_leaf=np.arange(1, 6)  } ]  clf = GridSearchCV(GradientBoostingClassifier(), param_grid, scoring=scoring, cv=10, refit='AUC', return_train_score=True) | **conventional model:**  0.847-0.883  **conventional + GRS model:**  0.877-0.892 | **conventional model:**  0.851  n_estimators=30  max_depth=4  **conventional + GRS model:**  0.885  n_estimators=80  max_depth=4  min_samples_leaf=2 |

**Supplementary table 6** Comparison of type 2 diabetes risk prediction models using four classifiers

| **Classifier** | **CPH** | **ANN** | **RF** |
| --- | --- | --- | --- |
| **conventional model, ΔAUC (*P* value)^a^** |  |  |  |
| - **ANN** | 0.002 (0.846) |  |  |
| - **RF** | 0.028 (0.052) | 0.027 (0.052) |  |
| - **GBM** | **0.037 (<0.001)** | **0.035 (0.003)** | 0.008 (0.398) |
| **conventional + GRS model, ΔAUC (*P* value)^a^** |  |  |  |
| - **ANN** | 0.003 (0.566) |  |  |
| - **RF** | **0.046 (0.006)** | **0.043 (0.004)** |  |
| - **GBM** | **0.070 (<0.001)** | **0.067 (0.001)** | 0.024 (0.133) |
| Bold values represent P < 0.05. *Abbreviations: CPH, Cox proportional hazards regression model; ANN, artificial neural network; RF, random forest; GBM, gradient boosting machine; GRS, genetic risk score; AUC, the area under receiver operating characteristic curve.*  *^a^ΔAUC was the difference of AUCs among conventional-genetic-combined models and conventional model.* | | | |
